# Variants in inflammation-related genes influence the outcomes of physical exercise programs: A longitudinal study in Brazilian adolescents with overweight and obesity

**DOI:** 10.1590/1678-4685-GMB-2023-0211

**Published:** 2024-11-22

**Authors:** Ana Cláudia M.B. Gomes Torres, Neiva Leite, Ricardo Lehtonen Rodrigues de Souza, Juliana Pizzi, Gerusa Eisfeld Milano-Gai, Leilane Lazarotto, Luciane Viater Tureck, Lupe Furtado-Alle

**Affiliations:** 1 Universidade Federal do Paraná (UFPR), Departamento de Genética, Laboratório de Polimorfismos e Ligação, Curitiba, PR, Brazil.; 2 Universidade Federal do Paraná (UFPR), Departamento de Educação Física, Curitiba, PR, Brazil.

**Keywords:** Polymorphisms, homeostasis, inflammatory, cytokines, physical exercise

## Abstract

The expansion of adipose tissue, characteristic of obesity, releases inflammatory cytokines, leading to metabolic disorders. Physical activity, on the other hand, promotes fat loss and changes inflammatory profile. This study aimed to investigate the associations of 20 gene variants (*TLR2, TLR4, IL1B, IL6, NFKB1, TNF, NFKBIA, NLRC4, CARD8* and *NEK7*) with anthropometric and biochemical changes induced by physical exercise programs. Thus, 58 children and adolescents participated of the 12-week exercise programs. Parameters were collected before and after programs: body mass index, body fat percentage, LDL-C, HDL-C, triglycerides, total cholesterol, insulin, glucose, HOMA-IR and QUICKI. Changes in these parameters were calculated (final - initial measurements) for subsequent analyses. Linear regression analyses were performed to investigate associations between genotypes and changes in the analyzed parameters. We found associations between 14 variants in nine genes with anthropometrical and biochemical outcomes. Observing the distribution of the sample, the groups of individuals who responded less in relation to body fat and TG levels concentrated the highest scores of polygenic indexes as a result of a greater number of risk variants. In conclusion, some genotypes related to the inflammatory profile provided less favorable anthropometrical and biochemical outcomes in response to physical exercise programs.

## Introduction

Obesity is characterized by excess adipose tissue accumulation due to increased food ingestion, physical inactivity, and genetic susceptibility (Lister et al., 2023). According to World Health Organization (2024), more than 340 million children and adolescents (5 to 19 years old) were obese or overweight in 2016, and 39% of children under five were overweight or obese in 2020. Obesity leads to a state of chronic low-grade inflammation caused by the infiltration of macrophages in adipose tissue (Hotamisligil et al., 1995; Choe et al., 2016). The main hallmark of obesity-induced inflammation is a change of macrophage profile (M1 pro-inflammatory profile) responsible for releasing pro-inflammatory cytokines leading to disruption of metabolic homeostasis (Castoldi et al., 2016; Callegari et al., 2023). This dysfunction contributes to comorbidities such as type 2 diabetes, atherosclerosis, and non-alcoholic fatty liver disease (Li X *et al.*, 2023).

Some components activate the inflammatory process in obesity, recruiting macrophages to adipose tissue (Castoldi et al., 2016; Callegari et al., 2023). Free fatty acids (FFA) released by the lipolysis process, saturated fatty acids (SFA) from the diet, modified low-density lipoprotein (modified LDL), and lipopolysaccharides (LPS) from gram-negative bacteria of gut flora are some examples (Li X et al., 2023). Innate immune receptors recognize these components, TLR (toll-like receptors) and NLR (nod-like receptors), also called inflammasome in adipose tissue and macrophages, as an endogenous pathogen-associated molecular pattern (PAMPs) and endogenous damage-associated molecular pattern (DAMPs) (Akter, 2022; Wang et al., 2023). 

Mainly TLR2 (toll-like receptor 2) and TLR4 (toll-like receptor 4) recognize PAMPs or DAMPs, activate NF-κB (nuclear factor kappa B) signaling and induce the release of pro-inflammatory cytokines, such as TNFα (tumor necrosis factor) and IL-6 (interleukin 6) in adipose tissue. Also, after TLR activation, it triggers the process of inflammasome assembling. NLRP3 (NLR family pyrin domain-containing 3) inflammasome is the best-known player related to obesity-induced inflammation (Fernandes et al., 2020; Wu et al., 2020). Once activated, the NLRP3 inflammasome leads to the production of pro-inflammatory cytokines interleukin 1 beta (IL-1β) and interleukin 18 (IL-18) in a process dependent on caspase-1 (Yang et al., 2019). Besides NLRP3, other inflammasomes have been related to metabolic disorders, including NLRP1 (Murphy et al., 2016) and NLRC4 (Xu et al., 2022). Releasing pro-inflammatory cytokines contributes to insulin resistance, atherosclerosis, and other metabolic dysfunction (Suzuki, 2019). 

In summary, a break of metabolic homeostasis, caused by an excess of nutrient intake and associated obesity, increase activation of the immune response, mainly in adipose tissue, releasing inflammatory markers. In this context, tissue inflammation called metainflammation leads to neurometabolic disorders (Russo et al., 2021). The interplay between immunology and metabolism has been studied in different contexts, and evidence about the inextricable relationship between the immune state and metabolism has been accumulating, delimiting immunometabolism as a study area (Lercher et al., 2020; Chavakis, 2022).

On the other hand, regular physical exercise improves metabolic parameters (lipid, glucose levels, and anthropometrical parameters) and reduces low-grade inflammation observed in metabolic diseases, including obesity (Gonzalo-Encabo et al., 2021). In addition, it promotes beta-adrenergic signaling promoting attenuation of the inflammatory profile (Simpson et al., 2021). Despite the known therapeutic role of physical exercise on obesity and other metabolic diseases, the possible mediating role of immune system elements in response to exercise still lacks studies. Thus, we aimed to investigate whether anthropometrical and biochemical outcomes of 12 weeks of physical exercise are related to variants in genes coding for immune response components (*TLR2, TLR4, IL1B, IL6, TNF, NFKB1, NFKBIA, NLRC4, CARD8,* and *NEK7*) in overweight and obese children and adolescents.

## Subjects and Methods

### Study design

In this study, we analyzed three independent samples together. Each sample was composed of a published independent study, which aimed to investigate the effects associated with each physical exercise modality. Thus, each group was submitted to one of the following exercise programs: HIIT (High-Intensity Interval Training) was conducted by Pizzi and coworkers (Pizzi et al., 2017) or land-based aerobic exercise by Milano et al., 2013 (Milano *et al.,* 2013). 

The same procedures were applied in each study. Children and adolescents were recruited in public schools in Paraná, Brazil, and the inclusion criteria were medical liberation for exercise practice, and not use of weight and lipid levels control drugs. Each physical exercise program was applied for 12 weeks, with three weekly sessions. Details of the exercise program are described below. This study was approved by the ethics committee of Federal University (protocol number: 2460.067/2011) and Pontifical Catholic University of Parana’s Institutional Ethics Board (IEB approval number: 0005306/11). Informed consent term was obtained from parents or the legal responsibility of all participants. 

Regardless of the physical exercise modality, only individuals who attended the pre and post-intervention data collection were considered for the longitudinal analyses performed in this study. Thus, the total sample comprised 58 children and adolescents (33 boys and 25 girls), of which 47 were obese: BMI- Z score >+2 SD, and 11 were overweight: BMI-Z score >+1 SD analyzed. The mean age was 12.04±1.64 years old. 

### Physical exercise programs

The exercise programs were carried out as described below:

HIIT (n=25): each session constituted 45 minutes duration with two sets of activities. Each set consisted of warm-up exercises, running for 30 seconds at 100% speed peak effort and walking for 60 seconds at 50% peak velocity (active recovery period). There was a rest period of 4 minutes between sets. The training progressed weekly by adding time to the running exercises and decreasing rest time. In week 1, the set was composed by 4 x the 30s/60s; in week two by 5 x 30s/60s; in week three by 6 x 30s/60s; in week 4 and 5 by 7 x 30s/60s; week 6 to 9 by 8 x 30s/45s (recovery period was decreased) and week 10 to 12 by 8 x 30s/30s (Pizzi et al., 2017)

Land-based aerobic exercise (n=33): Each session comprised 45 minutes of walking, 45 minutes of indoor cycling, and 20 minutes of stretching. Indoor cycling and walking started in 35% and 55% of rate heart reserve (RHR), increasing to 45% and 65% in the 5^th^ to the 8^th^ week and reaching 55% and 75% in the 9^th^ to 12^th^ week (Milano et al., 2013). 

### Data collection

Weight and height were collected to calculate the BMI-Z score (body mass index for age - Z score), and the body fat percentage (fat) was estimated using the bioimpedance technique. Peripheral blood samples were collected from all individuals to proceed with the DNA extraction and measurement of the following biochemical variables: HDL-cholesterol (HDL-C), LDL-cholesterol (LDL-C), triglycerides (TG), total cholesterol (TC), insulin and glucose levels. Homeostatic Model Assessment for Insulin Resistance (HOMA-IR) was calculated by the formula (glucose (nmol/L) x insulin (µU/mL)/22.5) (Matthews et al., 1985). Quantitative Insulin Sensitivity Check Index (QUICKI) was calculated using 1/(log fasting glucose (mmol/L) + log fasting insulin (µU/mL)) (Katz et al., 2000). All variables were collected initially and at the end of the physical exercise programs.

### Polymorphism selection and genotyping

Candidate genes were selected based on their inflammatory pathway role. Polymorphisms in the selected genes were chosen based on their high LD (linkage disequilibrium) (D’>0.8), representing, thus, a block of SNPs (tag SNPs). Furthermore, another criterion was the existence of SNPs with relevant associations in candidate genes association and or GWAS (Genome Wide Association Studies) in the linkage disequilibrium blocks (Table 1). All SNPs selected had minor alleles at a frequency (MAF) of at least 10%, and the selected tag SNPs did not capture all the variation existing in the genes. 

**Table 1 -:** Characteristics of the SNPs included in this study.

Gene	SNP	Nucleotide substitution	Location	MAF*	Type
*TLR2*	rs13105517	c.-1110 G>A	5’UTR	0.28 (A)	Tag SNP
*TLR2*	rs3804099	c.597 T>C (p.Asn199=)	Coding sequence	0.46 (C)	Literature
*TLR4*	rs1927911	c.-147-423A>G	Intron variant	0.28 (A)	Tag SNP
*TLR4*	rs1554973	c.260+9805T>C	3’UTR	0.23 (C)	Literature
*NLRC4*	rs212704	c.2783-514C>T	Intron variant	0.39 (C)	Tag SNP
*NLRC4*	rs455060	c.1824G>A (p.Ala608=)	Coding sequence	0.32 (G)	Tag SNP
*NLRC4*	rs385076	c.-119+805T>C	5’UTR	0.32 (T)	Literature
*CARD8*	rs1968440	c.129-2125A>G	3’UTR	0.11 (A)	Tag SNP
*CARD8*	rs6509366	c.59+642G>A	Intron variant	0.29 (A)	Tag SNP
*CARD8*	rs7258674	c.-252+21A>G	Intron variant	0.40 (A)	Tag SNP
*NEK7*	rs6671879	c.29+14532A>G	Intron variant	0.39 (G)	Tag SNP
*NFKB1*	rs3774932	c.-54+1248A>T	Intron variant	0.40 (A)	Tag SNP
*NFKB1*	rs3755867	c.1750-979A>G	Intron variant	0.30 (G)	Tag SNP
*NFKBIA*	rs3138053	c.-1004A>G	Promoter region	0.32 (C)	Tag SNP
*NFKBIA*	rs696	c.*126C>T	3´UTR	0.37 (T)	Literature
*IL6*	rs2069845	c.2783-514C>T	Intron variant	0.39 (C)	Tag SNP
*TNF*	rs1800629	c.-488G>A	Promotor	0.18 (A)	Literature
*TNF*	rs915654	c.-1736T>A	Intergenic	0.33 (A)	Literature
*IL1B*	rs1143634	c.315G>A	Coding sequence	0.23 (A)	Tag SNP
*IL1B*	rs3917356	c.99+780C>T	Intron variant	0.44 (T)	Tag SNP
*IL1B*	rs16944	c.-598A>G	5’UTR	0.34 (A)	Literature

Legend: * MAF: minor allele frequency-based in CEU population (Utah residents with Northern and Western European Ancestry. UTR: untranslated region; Tag SNP: polymorphism selected based on linkage disequilibrium (LD); Literature: polymorphism previously associated with metabolic disorders.

Genomic DNA was extracted from peripheral blood by the salting-out method adapted from Lahiri and Nurnberger (Lahiri and Nurnberger 1991). DNA samples were diluted to a final concentration of 20 ng/µl. Genotyping was performed by Sequenom MassARRAY iPLEX platform (Gabriel et al., 2002). 

### Statistical analysis

Allelic and genotype frequencies were obtained by direct counting (Table S1). The chi-square test calculated the Hardy-Weinberg equilibrium. The continuous data were tested for normality by Kolmogorov-Smirnov with Lilliefors correction. Genotypes were evaluated for two interaction models, recessive and dominant. In the recessive model, the minor homozygotes were compared with common allele carriers (heterozygotes and homozygotes for common allele). The minor allele carriers (heterozygotes and homozygotes for the minor allele) were compared with common allele homozygotes in the dominant model. 

Means of the variables pre and post-exercise programs were compared by paired t-test for parametric variables and by Wilcoxon test for non-parametric variables (Table 2). The delta was obtained for all variables (calculated using final minus initial measures) to check the exercise programs outcomes according to genotypes. The deltas of means were compared between genotypes by independent t-test for parametric variables and Mann-Whitney for non-parametric variables. 

**Table 2 -:** Characteristics of the participants pre and post-exercise programs.

Variables	n	Pre-intervention mean±SD	Post-intervention mean±SD	P
BMI Z	58	2.72±0.76	2.70±0.79	0.6238
Fat (%)	43	40.07±8.55	36.24±9.02	0.0001
TC (mg/dL)	52	165.82±41.17	158±35.97	0.0259
LDL-C (mg/dL)	52	98.83±30.22	91.48±29.31	0.0259
HDL-C (mg/dL)	51	48.87±15.86	47.79±16.53	0.4531
TG (mg/dL)	52	106.88±59.32	107.78±61.63	0.5655
Insulin (µUI/mL)	50	17.28±14.71	13.49±7.71	0.0175
Glucose (mg/dL)	49	82.35±9.33	81.77±5.85	0.6499
HOMA-IR	49	3.29±2.60	2.55±1.56	0.0259
QUICKI	49	0.33±0.03	0.34±0.03	0.0259

Legend: BMI Z: BMI-Z score; Fat: body fat percentage; TC: total cholesterol; LDL-C: LDL-cholesterol; HDL-C: HDL-cholesterol TG: triglycerides; HOMA-IR: homeostasis model assessment (HOMA) indexes; QUICKI: Quantitative Insulin sensitivity Check Index; n: number of participants; SD: standard deviation; p-value, adjusted according to the Benjamini-Hochberg method from comparisons of means, paired t-Test or Wilcoxon test.

Linear regression analyses were modeled to analyze possible associations between genotypes and biochemical and anthropometrical outcomes. First, univariable linear regressions were used to refine possible associations between genotypes (independent variables) and exercise programs outcomes (delta - as dependent variable). Subsequently, using the significant results of these univariable linear regressions, multivariable linear regression models were adjusted for age, sex, and type of exercise. P-values were adjusted for multiple comparisons according to the Benjamini-Hochberg method (Benjamini and Hockberg, 1995).

In addition, polygenic scores were calculated through the PredictABEL package (Kundu et al., 2011). These indexes were obtained only for the characteristics (delta values) associated with three or more polymorphisms through the univariable linear regressions, thus, only for the fat and TG levels changes were obtained polygene scores. 

To transform these quantitative changes in fat and TG levels into binary results, the median of delta for these parameters was obtained. In this way, individuals could be classified according to their position in relation to these medians. For example, individuals with fat delta values below the median, that is, they lost little fat, were coded “zero”. Individuals above the median - lost more fat, received code “one”.

From this analysis, the polygenic index (polygenic risk score, PRS) was calculated for each individual in the sample. This calculation was based on the presence or absence of the genotype or risk allele for the SNPs that showed a significant association in the univariable regression. Each of these SNPs was weighted by a specific index, calculated as 1 minus the p value obtained from the univariable regression analysis corresponding to that allele or genotype. Thus, for each individual, the PRS calculation consists of the sum of the risk alleles and/or genotypes (count of all loci), weighted for each SNP by the index (1 minus the regression p-value).

The statistical significance adopted for all tests was 0.05 (5%). Analyses were performed in the R program. 

## Results

### Effects of the physical exercise programs regardless of the analyzed SNPs

The exercise programs were effective in improving the cardiometabolic profile of the adolescents, as follows: fat was reduced by 3.83%; TC was decreased by 4.7%, insulin was reduced by 21.9%, HOMA-IR reduced by 22.5%, and QUICKI increased by 3%, on the other hand, HDL-C reduced by 2.2% (Table 2). 

### Inflammation-related genes and the exercise programs outcomes

All SNPs were in Hardy-Weinberg equilibrium. Allelic and genotypic frequencies of the polymorphisms included in this study were described in Table S1. Significant associations between anthropometrical outcomes and biochemical outcomes and polymorphisms, corrected for age, sex, and type of exercise, were summarized in Table 3 and Table 4. Results of all comparisons are shown in Table S2. 

**Table 3 -:** Associations between genotypes and changes in body fat percentage and BMI Z score.

Gene	SNP	Variable	Genotypes	Delta±SD	P
*TLR4*	rs1927911	BMI Z	AA (n=4)	-0.06±0.23	0.0036
			GG+GA (n=50)	0.004±0.28	
*NFKB1*	rs3755867	Fat	GG (n=4)	-10.05±3.60	0.0204
			GA+AA (n=39)	-3.19±4.66	
		BMI Z	GG (n=7)	0.18±0.57	0.0471
			GA+AA (n=47)	-0.05±0.18	
*IL1B*	rs3917356	Fat	TT (n=8)	-0.70±4.5	0.0471
			CC+CT (n=35)	-4.54±4.84	
*NLRC4*	rs212704	Fat	CC (n=8)	-0.23±5.14	0.0883
			TT+CT (n=35)	-4.65±4.61	
	rs385076	Fat	TT (n=5)	1.72±5.48	0.0204
			CC+CT (n=38)	-4.56±4.47	
	rs455060	Fat	GG (n=5)	1.00±4.49	0.0204
			AA+GA (n=38)	-4.46±4.55	

Legend: BMI Z*:* BMI-Z score*;* Fat: body fat percentage; n: number of participants; SD: standard deviation; p-value, adjusted according to the Benjamini-Hochberg method, from multiple regression analyses corrected for age, sex, and type of exercise, delta= final - initial measure.

**Table 4 -:** Associations between genotypes and changes in biochemical outcomes.

Gene	SNP	Variable	Genotypes	Delta±SD	p
***TLR2***	rs13105517	QUICKI	AA (n=5)	0.02±0.01	0.0364
			GG+GA (n=44)	0.004±0.02	
		HOMA-IR	AA (n=5)	-2.42±3.63	0.0322
			GG+GA (n=44)	-0.43±1.24	
	rs3804099	TG	CC (n=12)	29.16±88.20	0.0322
			TT+CT (n=40)	-7.58±40.13	
***TLR4***	rs1927911	HOMA-IR	AA (n=4)	-2.25±4.47	0.0471
			GG+GA (n=45)	-0.49±1.22	
***IL6***	rs2069845	TC	AA (n=28)	-1.87±22.77	0.0471
			AG+GG (n=24)	-14.76±20.57	
		LDL-C	AA (n=28)	-1.48±21.71	0.0322
			AG+GG (n=24)	-14.19±16.38	
		HDL-C	AA (n=27)	1.60±10.30	0.0640
			AG+GG (n=24)	-4.09±8.97	
***NFKBIA***	rs3138053	Glucose	TT (n=28)	1.76±8.93	0.0471
			CC+CT (n=21)	-3.70±7.88	
***NFKB1***	rs3755867	LDL-C	GG (n=6)	2.10±28.34	0.0471
			GA+AA (n=46)	-8.58±19.07	
***CARD8***	rs6509366	TG	AA (n=5)	54.8±97.68	0.0364
			GG+AG (n=47)	-4.83±48.14	
	rs7258674	HDL-C	AA (n=5)	-11.28±8.97	0.0322
			GG+AG (n=46)	0.54±9.27	
		TC	AA (n=5)	-26.42±23.07	0.1097
			GG+AG (n=47)	-4.93±21.26	
***NEK7***	rs6671879	Glucose	GG (n=10)	-7.6±7.21	0.0322
			AA+AG (n=39)	1.22±8.38	
		TG	AA (n=16)	28.14±88.15	0.0322
			GG+AG (n=36)	-11.20±27.85	

Legend: TG: triglycerides (mg/dL); TC: total cholesterol (mg/dL); LDL-C: LDL-cholesterol (mg/dL); HDL-C: HDL-cholesterol (mg/dL); QUICKI: Quantitative Insulin Sensitivity Check Index; HOMA-IR: homeostasis model assessment (HOMA) indexes; QUICKI: Quantitative Insulin sensitivity Check Index; n: number of participants; SD: standard deviation; p-value, adjusted according to the Benjamini-Hochberg method, from multiple regression analyzes corrected for age, sex, and type of exercise, delta= final - initial measure.

We found associations between polymorphisms in the *TLR4, NFKB1, IL1B*, and *NLRC4* genes with BMI Z score and fat. AA homozygotes of the rs1927911 (*TLR4*) showed a decrease in BMI Z compared to G allele carriers. GG homozygotes of the rs3755867 (*NFKB1)* had a higher fat loss when compared with the A allele carriers. On the other hand, A allele carriers had a decrease in BMI Z compared to the GG homozygotes individuals. C allele carriers of rs3917356 (*IL1B*), C allele of rs385076 (*NLRC4),* and A allele of rs455060 (*NLRC4*) had a higher fat loss when compared with TT, TT, and GG genotypes, respectively (Table 3).

Significant associations between biochemical outcomes and polymorphisms are summarized in Table 4. 

We found associations between glucose and predictors of insulin sensitivity with some investigated polymorphisms: AA homozygotes of rs13105517 (*TLR2*) and AA homozygotes of rs1927911 (*TLR4)* had improvement in insulin sensitivity according to the HOMA-IR index decreasing. In addition, AA homozygotes (rs13105517) (*TLR2*) also increased QUICKI values. C allele carriers of rs3138053 (*NFKBIA*) and GG homozygotes of rs6671879 (*NEK7*) had a higher reduction in glucose levels (Table 4). 

Regarding the lipid profile, T allele carriers of rs3804099 (*TLR2),* the G allele of rs6509366 (*CARD8*), and the G allele of rs6671879 (*NEK7*) were associated with a reduction of TG levels. Conversely, CC, AA, and AA homozygotes had increased TG levels. G allele carriers of rs2069845 (*IL6*) presented a higher reduction of TC and LDL-C levels than AA homozygotes. G allele carriers of rs7258674 (*CARD8)* exhibited increased HDL-C levels compared with AA homozygotes individuals. A allele carriers of rs3755867 (*NFKB1*) had decreased levels of LDL-C in comparison with GG homozygotes (Table 4). 

Polygenic indexes were performed only for the outcomes associated with three or more polymorphisms. Thus, the analyses were conducted only for the fat and TG. 

The risk allele or genotypes were: TT genotype of rs3917356 (*IL1B)*, A allele of rs3755867 (*NFKB1*), CC genotype of rs212704 (*NLRC4*), TT genotype of rs385076 (*NLRC4*) and GG genotype of rs455060 (*NLRC4*), associated with worse performance in relation to fat loss in response to the physical exercise programs. 

**Figure 1 -:**
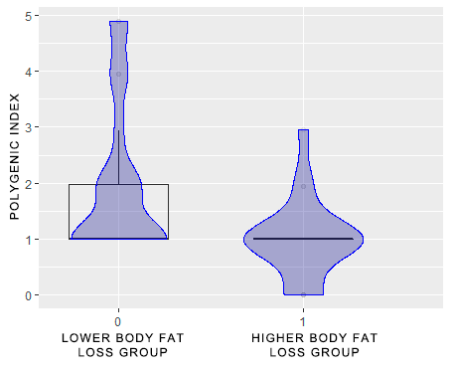
Sample distribution according to the relationship between the polygenic index and the body fat change in response to physical exercise programs. A polygenic index was assigned for each individual, consisting of the number of risk genotypes or alleles weighted by the p-value from univariable regression for fat change. Five variants were associated with poorer fat loss performance after the physical exercise programs: TT genotype of rs3917356 (*IL1B*), A allele of rs3755867 (*NFKB1*); CC genotype of rs212704 (*NLRC4*); TT genotype of rs385076 and GG genotype of rs455060 (*NLRC4*). Individuals were divided into two groups, according to the individual body fat delta and the median of the body fat delta: individual body fat delta below the median of the body fat delta - lower body fat loss group; individual body fat delta above the median of the body fat delta - higher body fat loss group.

Figure 1 shows the sample distribution in relation to fat change and the polygenic index. Firstly, the individuals were grouped according to the median of fat delta: participants with fat delta below the median were part of the “lower body fat loss group”; participants with fat delta above the median were part of the “higher body fat loss group”. From the distribution of individuals in these two groups in relation to the polygenic index, it is possible to observe that the individuals´ scores varied between one and five among those who lost less fat. Already, the scores of individuals who lost more fat varied between zero and three. 

**Figure 2 -:**
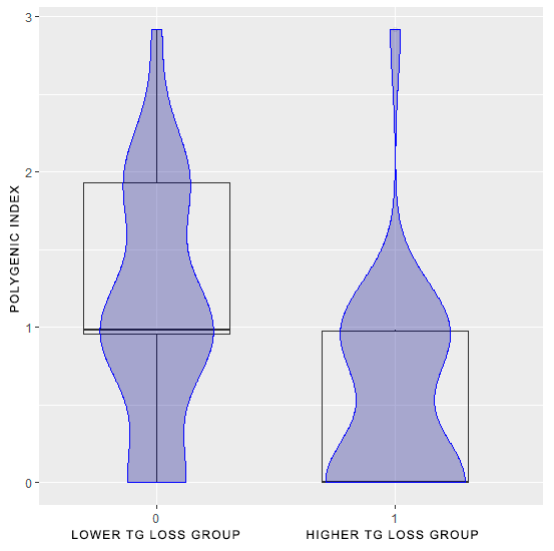
Sample distribution according to polygenic index and the TG change in response to physical exercise programs. A polygenic index was assigned for each individual, consisting of the number of risk genotypes or alleles weighted by the p-value from univariable regression for TG change. Three variants were associated with TG change after the physical exercise programs: T allele of rs3804099 (*TLR2)*, G allele of rs6509366 (*CARD8*), and G allele of rs6671879 (*NEK7*). Individuals were divided into two groups, according to the individual triglycerides delta and the median of the triglycerides delta: individual triglycerides delta below the median of the triglycerides delta - lower triglycerides loss group; individual triglycerides delta above the median of the triglycerides delta - higher triglycerides loss group. Legend: TG: triglycerides.

Figure 2 shows the same sample distribution, but in relation to triglycerides change. Individuals were grouped according to the median of TG delta: participants with TG delta below the median were part of the “lower TG loss group”; participants with TG delta above the median were part of the “higher TG loss group.” From the distribution of individuals in these two groups in relation to the polygenic index, it is possible to observe that the individuals´ scores varied between zero and three among those whose decrease in TG levels was smaller. The scores of individuals whose TG levels decreased the most also varied between zero and three, however, the majority of individuals presented scores between zero and one. 

Table 5 summarizes significant associations between biochemical and anthropometrical parameters found in this study. 

**Table 5 -:** Summary of associations found in this study.

		Anthropometric		Biochemical						
GENE	SNP	BMI-Z	FAT	HOMA.IR	QUICKI	GLUCOSE	LDL.C	HDL.C	TC	TG
*TLR2*	rs13105517			AA▼+ GG+GA▼	AA▲+ GG+GA▲	.	.	.	.	.
	rs3804099	.	.	.	.	.	.	.	.	CC▲ TT+CT▼
*TLR4*	rs1927911	AA▼ GG+GA▲	.	AA▼+ GG+GA▼	.	.	.	.	.	.
	rs1554973		.	.	.	.	.	.	.	.
*IL6*	rs2069845	.	.	.	.	.	AA▼ AG+GG▼+		AA▼ AG+GG▼+	.
*NFKB1*	rs3755867	. GG▲ GA+AA▼	GG▼+ GA+AA▼	.	.	.	GG▲ GA+AA▼	.	.	.
*NFKBIA*	rs3138053	.	.	.	.	TT▲ CC+CT▼	.	.	.	.
*IL1B*	rs3917356	.	TT▼ CC+CT▼+	.	.	.	.	.	.	.
*NLRC4*	rs212704		CC▼* TT+CT▼+	.	.	.	.	.	.	.
	rs385076	.	TT▲ CC+CT▼	.	.	.	.	.	.	.
	rs455060		GG▲ AA+GA▼	.	.	.	.	.	.	.
*CARD8*	rs6509366	.	.	.	.	.	.	.	.	AA▲ GG+AG▼
	rs7258674	.	.	.	.	.	.	AA▼ GG+AG▲	.	.
*NEK7*	rs6671879	.	.	.	.	GG▼ AA+AG▲	.	.	.	AA▲ GG+AG▼

Legend: BMI Z*:* BMI-Z score*;* Fat: body fat percentage; TC: total cholesterol; LDL-C: LDL-cholesterol; HDL-C: HDL-cholesterol TG: triglycerides; HOMA-IR: homeostasis model assessment (HOMA) indexes; QUICKI: Quantitative Insulin sensitivity Check Index. Green arrows: decrease or improvement in the parameters. Green arrows with plus: higher decrease of parameters compared with another genotype. Red arrows: increase or worsen in the parameters; Asterisk: polymorphisms without association in multivariable linear regression but included in the polygenic index.

## Discussion

To the best of our knowledge, the present study is the first to investigate the role of genes related to inflammatory pathways in physical exercise outcomes of obese children and adolescents. Different alleles and genotypes of the investigated genes were associated with different responses in aspects of metabolism, reflected in the participants’ responsiveness variation regarding important parameters, such as fat percentage, glucose and insulin sensitivity, and lipid profile components. 

Obesity promotes chronic inflammation and oxidative stress, which induces several metabolic disorders, such as insulin resistance, hyperlipidemia, and complications (Jimenez-Duran and Triantafilou, 2021). The increase in white adipose tissue is generally associated with strong infiltration and polarization towards M1-like macrophages, a phenotype that promotes a pro-inflammatory Th1 response (Flaherty *et al.,* 2019; Li C et al., 2020; Makowski et al., 2020). This predominance of the pro-inflammatory macrophage phenotype is associated with higher TNF-α, IL-6, and IL-1β expression (Parisi et al., 2018). 

In this context, physical exercise is an important strategy to promote health improvement, helping to decrease inflammatory markers and maintain normal lipid and glucose levels. The skeletal muscle contractions induce responses in the whole body, and many of these responses are due to its anti-inflammatory effect (Goh et al., 2016; Krüger et al., 2014).

Regular physical exercise enhances adipokine release, such as leptin and adiponectin, and decreases pro-inflammatory cytokines, including TNFα (34-36), followed by an increase in IL-6 release (Pedersen, 2017). Besides that, physical exercise leads to skeletal muscle release of myokines, such as irisin, which downregulates pro-inflammatory cytokines, particularly TLR4 activity (Mazur-Bialy et al., 2017; Suzuki, 2019). In addition, the inflammasome, an essential complex of the immune system associated with metabolic inflammation, was suppressed by physical exercise, lowering the release of pro-inflammatory cytokines (Bartlett et al., 2018; Lee et al., 2018; Rada et al., 2018; Jablonski et al., 2020). 

Our results indicate a set of alleles and genotypes of the polymorphisms in inflammation-related genes associated with relatively poorer performance in response to physical exercise programs, especially concerning changes in fat and triglyceride levels. Regarding this set of variants, most of these alleles or genotypes were individually previously associated with less favorable metabolic or immunological characteristics or even, in some cases, demonstrated an effect on gene expression (Ko et al., 2009; Fang et al., 2013; Zeller et al., 2015; Tak et al., 2018; Gomes Torres et al., 2019; Xu et al., 2020; Fei et al., 2022). As observed by Zeller and colleagues, the functional effect of C allele (rs385076 *NLRC*4) with increase of IL-18 levels in cardiovascular disorders (Zeller *et al.,* 2015), T allele of rs385076 was associated with autoimmune thyroid disease (Liu et al., 2020) and TT genotype with young onset of type 1 diabetes in a study conducted with Chinese population (Xu *et al.,* 2020). In addition, Xu and coworkers also found association with CC genotype of rs212704 (*NLRC4*) and lower post prandial C peptide, showing an impairment on islet function (Xu *et al.,* 2020).

Conversely, negative regulators of NLRP3, such as CARD8 are predicted to act as protective factor against exacerbation of inflammation response (Ito et al., 2014). Therefore, variants in *CARD8* related with its lower expression also was associated with autoimmune disorders including rheumatoid arthritis (Lee and Song 2023) and type 1 diabetes (Pang et al., 2021). In this context, NLRP3 inflammasome ablation in macrophages of 24-month-old mice increases visceral adipose tissue lipolysis through increased catecholamine levels, suggesting that inflammation plays a role in lipolytic effects (Camell et al., 2017). Considering these findings, inflammasome activation leads to the processing of pro-IL-1β into mature IL-1β, which is then secreted from the cell injuring the target organs. In a seminal study Luotola et al. (2009) associated variants in *IL1B* (haplotype ACG of rs1143634, rs3917356, and rs16944 respectively) with impairs of glucose metabolism and diabetes in Finland population. Effect of chronic inflammation on health is critical because cytokines released blunts the positive metabolic improvements to exercise. 

Metainflammation and physical exercise have opposite effects on the lipolysis process. Physical exercise is one of the most potent stimuli for promoting lipolysis. Lipolysis is a process that basically consists of the breakdown of lipids in adipose tissue and the release of fatty acids into circulation, directly impacting body fat and serum triglyceride levels. On the other hand, metainflamation is acutely catabolic and can stimulate the reverse path, adipogenesis, and impair catecholamine-induced lipolysis (Della Guardia and Wang, 2023). 

Catecholamines mainly mediate the lipolysis process stimulated by physical exercise. Some researchers have long described the situation of catecholamines resistance in individuals with obesity; that is, an inability of adipocytes from individuals with obesity to respond to catecholamines to the same degree as those without obesity (Goldberg and Gordon, 1964; Connacher et al., 1991; Jensen, 1991; Martin and Jensen, 1991). Catecholamine sensitivity may be one of the mechanisms responsible for the variation in response to physical exercise programs observed in our study since the inflammatory profile has been described as one of the contributing factors for this condition. In this sense, the tumor necrosis factor-alpha (TNF-α), one of the main pro-inflammatory cytokines, acts by decreasing the *ADBR3* gene expression, which encodes the β3-adrenoceptor (β3-AR), responsible for mediating the catecholamine-lipolysis action (Hadri et al., 1997; Valentine et al., 2022). Indeed, Choi et al. (2015), identified repression of stimulated lipolysis and thermogenesis in a mouse model of a high-fat diet (HFD) - induced obesity, compared to control animals. In parallel, when investigating changes in global adipose tissue gene expression profiles in these animals, they identified that most genes up-regulated by HFD were related to immune and inflammatory responses. In contrast, genes involved in lipolytic, fatty acid oxidation, and thermogenic pathways, including Adrb3, were down-regulated (Choi *et al.,* 2015). Valentine and collaborators recently suggested that in an obese state, the β3-AR expression is already reduced due to metainflammation (Valentine *et al.,* 2022). 

In this sense, less favorable inflammatory profiles, such as those that may be associated with the genetic variants that conferred poorer fat and triglyceride levels loss after the physical exercise programs in our study, may, at least initially, benefit less from the effects of physical exercise, due to a more pronounced situation of resistance to catecholamines.

The current study has some important limitations. Firstly, the small sample size obtained for the study, which reduces statistical power and, therefore, the possibility of observing relevant associations. The adherence and attendance of participants in physical intervention programs are very difficult parameters to obtain, especially with child and adolescent participants. Such factors were decisive for the small sample size of the study. Another factor that may have limited our results was the duration of the physical exercise programs. Given the difficulty in keeping participants until the end of the program, extending its application beyond 12 weeks was unfeasible, however, studies with longer intervention times are necessary to verify the persistence of the results found in our study, as well as verifying other associations that may derive from long-term effects of exercise programs. The lack of measurement of inflammatory markers in the sample was also limiting, since the investigation of correlations between genotype and inflammatory profile of participants could be relevant in the context of the study.

## Conclusion

In conclusion, we verified that the physical exercise programs effectively reduced anthropometrical and metabolic parameters. Variants in *TLR2, TLR4, IL1B, IL6, NFKB1, NFKBIA, NLRC4, CARD8*, and *NEK7* genes influenced the responsiveness of individuals to physical exercise programs regarding anthropometrical and metabolic outcomes. The combination of the *IL1B, NFKB1, NLRC4* and *TLR2, CARD8, and NEK7* gene variants seems to be relevant to the fat and TG levels response stimulated by physical exercise programs. In this way, variation in genes related to inflammatory pathways has been shown to influence the level of responsiveness to physical exercise programs applied.

## Supplementary material

Table S1 - Allelic and genotypic frequencies of variants analyzed.

Table S2 - Results of means comparison and multivariable regression.
