# Supplementary material for: Variants in inflammation-related genes influence the outcomes of physical exercise programs: A longitudinal study in Brazilian adolescents with overweight and obesity
Source: Genet Mol Biol. 2024 Nov 22;47(4):e20230211. doi: 10.1590/1678-4685-GMB-2023-0211 (PMC11616735; doi:10.1590/1678-4685-GMB-2023-0211)
Supplement: Table S1 - [file 1415-4757-GMB-47-04-e20230211-s1.pdf]

Genetics and Molecular Biology
https://doi.org/10.1590/1678-4685-GMB-2023-0211

Supplementary Material to "Variants in Inflammation-related genes influence the outcomes of physical exercise programs: A longitudinal study in Brazilian adolescents with overweight and obesity"

Table S1 - Allelic and genotypic frequencies of variants analyzed.

Gene	SNP	Nucleotide change	MAF	Genotypes		
TLR2	rs13105517	G>A	A (0.34)	GG<br>23 (0.40)	GA<br>30 (0.52)	AA<br>5 (0.09)
TLR2	rs3804099	T>C	T (0.50)<br>C (0.50)	TT<br>13 (0.22)	TC<br>32 (0.55)	CC<br>13 (0.22)
TLR4	rs1927911	G>A	A (0.38)	GG<br>23 (0.40)	GA<br>30 (0.53)	AA<br>4 (0.07)
TLR4	rs1554973	T>C	C (0.28)	TT<br>29 (0.50)	TC<br>26 (0.45)	CC<br>3 (0.05)
IL6	rs2069845	A>G	G (0.28)	AA<br>32 (0.55)	AG<br>20 (0.34)	GG<br>6 (0.10)
NFKB1	rs3755867	A>G	G (0.35)	AA<br>24 (0.41)	AG<br>27 (0.47)	GG<br>7 (0.12)
NFKB1	rs3774932	G>A	A (0.38)	GG<br>20 (0.34)	GA<br>32 (0.55)	AA<br>6 (0.10)
NFKBIA	rs3138053	T>C	C (0.26)	TT<br>34 (0.59)	TC<br>18 (0.31)	CC<br>6 (0.10)
NFKBIA	rs696	C>T	T (0.33)	CC<br>26 (0.45)	CT<br>26 (0.45)	TT<br>6 (0.10)
TNF	rs1800629	G>A	A (0.11)	GG<br>46 (0.79)	GA<br>11 (0.19)	AA<br>1 (0.02)
TNF	rs915654	T>A	A (0.41)	TT<br>18 (0.32)	TA<br>31 (0.54)	AA<br>8 (0.14)
IL1B	rs1143634	G>A	A (0.15)	GG<br>42 (0.72)	GA<br>15 (0.26)	AA<br>1 (0.02)
IL1B	rs16944	G>A	A (0.45)	GG<br>19 (0.33)	GA<br>26 (0.45)	AA<br>13 (0.22)
IL1B	rs3917356	C>T	T (0.41)	CC<br>21 (0.36)	CT<br>26 (0.45)	TT<br>11 (0.19)
NLRC4	rs212704	T>C	C (0.42)	TT<br>18 (0.31)	TC<br>31 (0.53)	CC<br>9 (0.16)
NLRC4	rs385076	C>T	T (0.33)	CC<br>25 (0.43)	CT<br>28 (0.48)	TT<br>5 (0.09)
NLRC4	rs455060	A>G	G (0.38)	AA<br>20 (0.34)	AG<br>32 (0.55)	GG<br>6 (0.10)

Gene	SNP	Nucleotide change	MAF	Genotypes		
CARD8	rs6509366	G>A	A (0.31)	GG<br>27 (0.47)	GA<br>26 (0.45)	AA<br>5 (0.09)
CARD8	rs7258674	G>A	A (0.32)	GG<br>29 (0.50)	GA<br>21 (0.36)	AA<br>8 (0.14)
NEK7	rs6671879	A>G	G (0.47)	AA<br>17 (0.29)	AG<br>28 (0.48)	GG<br>13 (0.22)

Legend: MAF: Minor Allele Frequency.
