# Supplementary material for: Variants in inflammation-related genes influence the outcomes of physical exercise programs: A longitudinal study in Brazilian adolescents with overweight and obesity
Source: Genet Mol Biol. 2024 Nov 22;47(4):e20230211. doi: 10.1590/1678-4685-GMB-2023-0211 (PMC11616735; doi:10.1590/1678-4685-GMB-2023-0211)
Supplement: Table S2 - [file 1415-4757-GMB-47-04-e20230211-s2.pdf]

Genetics and Molecular Biology
https://doi.org/10.1590/1678-4685-GMB-2023-0211

Supplementary Material to "Variants in Inflammation-related genes influence the outcomes of physical exercise programs: A longitudinal study in Brazilian adolescents with overweight and obesity"

Table S2 - Results of means comparison and multivariable regression.

Gene	SNP Genotype	BMI-Z score delta ±SD	Fat delta ±SD	Glucose delta ±SD	TC delta ±SD	HDL-C delta ±SD	LDL-C delta ±SD	TG delta±SD	Insulin delta±SD	QUICKI delta±SD	HOMA delta±SD
TLR2	rs13105517										
	AA	0.15±0.71	-8.20±3.56	-4.10±10.57	-24.2±22.70	-1.32±8.79	-19.26±11.96	-23.10±17.19	-10.55±14.20	0.02±0.01	-2.42±3.63
	GG+GA	-0.03±0.18	-3.50±4.93	-0.17±8.68	-6.08±22.03	-1.05±10.23	-6.08±20.66	3.45±58.32	-3.03±8.34	0.004±0.02	-0.43±1.24
	p[1]	0.7809	0.6031	0.7565	0.5060	0.9814	0.6535	0.6535	0.6535	0.3629	0.6031
	p[2]	0.5600	0.5600	0.6570	0.5487	0.8428	0.7080	0.5600	0.4208	0.3093	0.2504
TLR2	rs3804099										
	CC	-0.04±0.17	-5.65±4.94	-2.18±10.88	-9.33±17.76	-0.45±10.25	-12.99±21.06	29.16±88.20	-3.78±7.38	0.01±0.01	-0.78±1.55
	TT+CT	-0.015±0.29	-3.27±4.91	-3.27±4.91	-7.37±23.95	-3.09±9.38	-5.66±20.01	-7.58±40.13	-3.79±9.70	0.004±0.02	-0.59±1.74
	p[1]	0.9761	0.6535	0.8175	0.9709	0.7689	0.9761	0.7098	0.7689	0.6535	0.7565
	p[2]	0.9664	0.5600	0.6570	0.8815	0.8815	0.5146	0.2504	0.9841	0.7224	0.8815
TLR4	rs1554973										
	CC	0.17±0.12	-3.80±3.53	-0.50±3.53	-16.5±14.84	1.6±3.39	-16.30±5.51	-36.50±24.74	2.47±5.19	-0.01±0.03	0.49±1.09
	TT+CT	-0.03±0.27	-3.83±5.09	-0.58±9.02	-7.47±22.81	-1.18±10.20	-6.99±20.60	2.39±56.66	-4.05±9.24	0.0069±0.01	-0.68±1.70
	p[1]	0.3450	0.9981	0.9981	0.8853	0.9429	0.9761	0.6535	0.6535	0.7689	0.6535
	p[2]	0.5928	0.9841	0.8815	0.6570	0.9560	0.6620	0.7239	0.6570	0.5427	0.6570
TLR4	rs1927911										
	AA	-0.06±0.23	-2.7±1.80	-7±7.78	-20.75±15.10	-0.20±6.38	-2.80±23.57	11.16±68.98	-2.26±5.93	0.0053±0.01	-0.38±1.23
	GG+GA	0.004±0.28	-3.94±5.18	-0.006±8.78	-7.03±22.97	-1.54±10.06	-10.31±17.75	-6.92±45.96	-4.80±10.79	0.006±0.02	-0.81±1.94
	p[1]	0.4683	0.9242	0.6523	0.6535	0.9709	0.7809	0.6535	0.9059	0.7350	0.9058
	p[2]	0.0840	0.8428	0.5600	0.5600	0.9589	0.5928	0.6765	0.6528	0.8288	0.3879

Gene	SNP Genotype	BMI-Z score delta ±SD	Fat delta ±SD	Glucose delta ±SD	TC delta ±SD	HDL-C delta ±SD	LDL-C delta ±SD	TG delta±SD	Insulin delta±SD	QUICKI delta±SD	HOMA delta±SD
IL-6	rs2069845										
	AA	0.030±0.35	-3.47±3.77	-1.22±9.71	-1.87±22.77	1.60±10.30	-1.48±21.71	-8.07±58.69	-3.47±8.94	0.004±0.019	-0.79±2.10
	AG+GG	-0.07±0.12	-4.20±6.04	0.28±7.69	-14.76±20.57	-4.09±8.97	-14.19±16.38	11.36±52,31	-4.19±9.65	0.008±0.02	-0.43±0.91
	p[1]	0.6535	0.9059	0.8683	0.3629	0.3629	0.3450	0.5060	0.8613	0.7689	0.9761
	p[2]	0.5600	0.7411	0.8815	0.3879	0.4716	0.2504	0.5928	0.9589	0.7411	0.7848
IL-1β	**rs3917356**										
	TT	-0.08±0.11	-0.70±4.5	1.55±9.49	-17.2±21.66	- 4.86±11.74	-12.43±18.93	1.85±24.77	0.12±3.21	-0.002±0.01	0.06±0.73
	CC+CT	-0.007±0.29	-4.54±4.84	-1.12±8.72	-5.59±22.4	-0.15±9.49	-6.14±20.62	0.67±61.55	-4.76±9.92	0.008±0.02	-0.81±1.82
	p[1]	0.7689	0.3629	0.7689	0.6535	0.6535	0.3450	0.8683	0.3629	0.5500	0.3629
	p[2]	0.7290	0.3879	0.8815	0.6528	0.6960	0.8364	0.9646	0.5146	0.5600	0.5250
NFKBIA	rs3138053										
	TT	-0.05±0.16	-3.14±5.08	1.76±8.93	-9.3±24.87	-0.98±11.90	-8.32±19.56	2.18±36.47	-2.09±4.48	0.05±0.01	-0.35±0.88
	CC+CT	0.01±0,36	-5.11±4.63	-3.70±7.88	-5.81±19.26	-1.20±6.77	-6.02±21.62	-0.85±76.27	-5.94±12.72	0.007±0.024	-1.01±2.36
	p[1]	0.9709	0.6535	0.3450	0.8853	0.9761	0.6535	0.6535	0.7809	0.6535	0.8248
	p[2]	0.6570	0.5250	0.3879	0.9841	0.9589	0.9589	0.9841	0.5600	0.9646	0.5600
NFKB1	rs3755867										
	GG	0.18±0.57	-10.05±3.60	-0.33±8.50	2.66±41.53	3.60±15.53	2.10±28.34	-18.50±42.33	-7.41±13.68	0.01±0.020	-1.74±3.56
	GA+AA	-0.05±0.18	-3.19±4.66	-0.61±8.99	-9.19±19.15	-1.70±9.13	-8.58±19.07	3.43±57.61	-3.29±8.48	0.005±0.02	-0.48±1.25
	p[1]	0.7242	0.2588	0.9761	0.6535	0.6535	0.6523	0.7565	0.9761	0.9059	0.9750
	p[2]	0.3879	0.2504	0.9315	0.5146	0.5600	0.3879	0.5600	0.5600	0.6528	0.5600

Gene	SNP Genotype	BMI-Z score delta ±SD	Fat delta ±SD	Glucose delta ±SD	TC delta ±SD	HDL-C delta ±SD	LDL-C delta ±SD	TG delta±SD	Insulin delta±SD	QUICKI delta±SD	HOMA delta±SD
CARD8	rs6509366										
	AA	-0.13±0.20	-1.30±0.44	-5.20±1.09	-4.80±18.43	-2.40±5.31	-11.84±10.74	54.8±97.68	-8.10±10.67	0.01±0.009	-1.86±2.06
	GG+AG	-0.01±0.2736	-3.95±5.05	-0.05±9.19	-8.14±23.06	-0.93±10.43	-6.87±21.07	-4.83±48.14	-3.31±9	0.005±0.02	-0.49±1.61
	p^1	0.8507	0.7874	0.6535	0.9709	0.9709	0.7689	0.5060	0.6535	0.7098	0.4683
	p^2	0.7018	0.6111	0.5600	0.8815	0.9589	0.8364	0.3093	0.6265	0.6765	0.5146
CARD8	rs7258674										
	AA	0.06±0.58	-3.48±6.17	2.83±9.64	-26.42±23.07	-11.28±8.97	-16.77±14.31	6.57±25.67	-5.81±13.80	0.03±0.024	-1.35±3.69
	GG+AG	-0.03±0.17	-3.89±4.79	-1.05±8.75	-4.93±21.26	0.54±9.27	-5.88±20.80	0.01±59.66	-3.51±8.55	0.006±0.020	-0.53±1.25
	p^1	0.9709	0.9761	0.7268	0.3450	0.2588	0.6535	0.7098	0.9761	0.7616	0.8853
	p^2	0.6139	0.7411	0.8364	0.5296	0.2504	0.8428	0.9664	0.6960	0.9664	0.5910
NLRC4	rs212704										
	CC	-0.0075±0.16	-0.23±5.14	-0.85±6.66	-8.60±28.30	-5.53±8.85	-9.12±13.51	10.85±44.25	-6.17±14.82	0.0041±0.016	-0.22±0.62
	TT+CT	-0.023±0.28	-4.65±4.61	-0.53±9.23	-7.68±21.71	-0.24±10.10	-7.03±21.39	-0.90±58.31	-3.33±7.85	0.006±0.02	-0.70±1.80
	p^1	0.7689	0.3450	0.9761	0.9761	0.6535	0.6535	0.8175	0.9761	0.9709	0.8853
	p^2	0.8815	0.5146	0.9664	0.9664	0.7411	0.7901	0.7901	0.8364	0.7411	0.8815
NLRC4	rs385076										
	CC	-0.07±0.13	-5.91±4.18	-0.04±9.30	-6.21±22.41	-0.35±11.71	-8.32±17.81	12.80±45.60	-1.64±4.03	0.0058±0.01	-0.31±0.75
	TT+CT	0.01±0.33	-1.84±4.91	-0.97±8.64	-9.09±22.93	-1.67±8.57	-6.57±22.33	-8.54±62.46	-5.34±11.39	0.006±0.02	-0.87±2.12
	p^1	0.7323	0.2588	0.9567	0.9059	0.9059	0.9908	0.3450	0.9074	0.9761	0.9761
	p^2	0.6139	0.2504	0.9664	0.5600	0.7411	0.7290	0.6528	0.5600	0.8815	0.5600

Gene	SNP Genotype	BMI-Z score delta ±SD	Fat delta ±SD	Glucose delta ±SD	TC delta ±SD	HDL-C delta ±SD	LDL-C delta ±SD	TG delta±SD	Insulin delta±SD	QUICKI delta±SD	HOMA delta±SD
NLRC4	rs455060										
	AA	-0.10±0.15	-6.17±4.87	-0.35±9.52	-8.44±24.70	-1.66±11.83	-9.36±19.27	11.47±49.52	-1.78±4.33	0.006±0.02	-0.34±0.77
	GG+GA	0.023±0.30	-2.44±4.55	-0.69±8.62	-7.49±21.67	-0.75±9.07	-6.28±21	-4.69±59.31	-4.82±10.78	0.005±0.02	-0.79±2.01
	p^1	0.3450	0.3450	0.9761	0.9761	0.9709	0.9761	0.6535	0.9709	0.9709	1.00
	p^2	0.5250	0.2504	0.9589	0.8685	0.9664	0.9589	0.7786	0.6528	0.8428	0.6570
NEK7	**rs6671879**										
	GG	-0.02±0.21	-4.11±5.18	-7.6±7.21	-1.95±35.31	2.29±14.85	-1.17±23.83	-23±24.21	-1.17±1.99	0.01±0.01	-0.40±0.48
	AA+AG	-0.02±0.28	-3.70±4.95	1.22±8.38	-9.22±18.63	-1.89±8.51	-8.68±19.43	6.59±60.18	-4.44±10.13	00.40±0.02	-0.69±1.88
	p^1	0.7565	0.9709	0.2588	0.7565	0.6535	0.7616	0.3629	0.8853	0.6535	0.9709
	p^2	0.9600	0.9156	0.0137	0.6057	0.5923	0.5097	0.2293	0.5163	0.2321	0.8579
	AA	-0.02±0.22	-2.28±5.66	1.66±8.14	-3.11±24.24	-2.80±9.71	-6.40±27.63	28.14±88.15	-5.15±12.10	0.001±0.02	-0.51±1.54
	GG+AG	-0.01±0.29	-4.36±4.68	-1.66±9.09	-9.91±21.75	-0.35±10.19	-7.77±16.49	-11.20±27.85	-3.08±7.35	0.008±0.20	-0.69±1.78
	p^1	0.9761	0.6535	0.6535	0.7268	0.7689	0.8175	0.3450	0.9059	0.7565	0.9761
	p^2	0.9589	0.5928	0.5146	0.8364	0.5600	0.9589	0.2504	0.7411	0.5894	0.8947

Legend: BMI Z: BMI-Z score; Fat: body fat percentage; TC: total cholesterol; LDL-C: LDL-cholesterol; HDL-C: HDL-cholesterol TG: triglycerides; HOMA-IR: homeostasis model assessment (HOMA) indexes; QUICKI: Quantitative Insulin sensitivity Check Index; n: number of participants; SD: standard deviation; p-values, adjusted according to the Benjamini-Hochberg method p^1 from comparisons of deltas t test or Mann-Whitney and p^2 from multiple regression analyses corrected for age, sex, and type of exercise; delta= final – initial measure.
